# Predictive three-biomarker panel in peripheral blood mononuclear cells for detecting hepatocellular carcinoma

**DOI:** 10.1038/s41598-024-58158-9

**Published:** 2024-03-29

**Authors:** Sara Fayazzadeh, Mahsa Ghorbaninejad, Amirhassan Rabbani, Javad Zahiri, Anna Meyfour

**Affiliations:** 1 Bioinformatics and Computational Omics Lab (BioCOOL), Department of Biophysics, Faculty of Biological Sciences, Tarbiat Modares University, Tehran, Iran; 2 Basic and Molecular Epidemiology of Gastrointestinal Disorders Research Center, Research Institute for Gastroenterology and Liver Diseases, Shahid Beheshti University of Medical Sciences, Tehran, Iran; 3 Department of Transplant and Hepatobiliary Surgery, Taleghani Hospital, Shahid Beheshti University of Medical Sciences, Tehran, Iran; 4 Department of Neurosciences, University of California, San Diego, CA USA

**Keywords:** Diagnostic markers, Cancer, Computational biology and bioinformatics, Transcriptomics

## Abstract

Hepatocellular carcinoma (HCC) ranks among the most prevalent cancers and accounts for a significant proportion of cancer-associated deaths worldwide. This disease, marked by multifaceted etiology, often poses diagnostic challenges. Finding a reliable and non-invasive diagnostic method seems to be necessary. In this study, we analyzed the gene expression profiles of 20 HCC patients, 12 individuals with chronic hepatitis, and 15 healthy controls. Enrichment analysis revealed that platelet aggregation, secretory granule lumen, and G-protein-coupled purinergic nucleotide receptor activity were common biological processes, cellular components, and molecular function in HCC and chronic hepatitis B (CHB) compared to healthy controls, respectively. Furthermore, pathway analysis demonstrated that “estrogen response” was involved in the pathogenesis of HCC and CHB conditions, while, “apoptosis” and “coagulation” pathways were specific for HCC. Employing computational feature selection and logistic regression classification, we identified candidate genes pivotal for diagnostic panel development and evaluated the performance of these panels. Subsequent machine learning evaluations assessed these panels’ performance in an independent cohort. Remarkably, a 3-marker panel, comprising RANSE2, TNF-α, and MAP3K7, demonstrated the best performance in qRT-PCR-validated experimental data, achieving 98.4% accuracy and an area under the curve of 1. Our findings highlight this panel’s promising potential as a non-invasive approach not only for detecting HCC but also for distinguishing HCC from CHB patients.

## Introduction

Primary liver cancer, approximately 75% histology of which is hepatocellular carcinoma (HCC)^1^, is the sixth most frequently occurring cancer and the third most common cause of cancer mortality in both sexes worldwide^2^. Most HCC patients are diagnosed at late or advanced stages when the treatment options are limited and it is hard to cure if not untreatable. HCC is a complex multifactorial disease. The most important HCC risk factors mainly include cirrhosis, chronic infection with hepatitis B virus (HBV), hepatitis C virus (HCV), alcoholic liver disease, and nonalcoholic steatohepatitis (NASH). Additional risk factors that are also known to increase the chance of developing HCC include tobacco smoking, aflatoxin-contaminated food intake, diabetes, obesity, genetic factors, and heredity^3,4^.

HCC is associated with poor prognosis and low survival rates. Therefore, early detection of HCC is beneficial for prolonging patient survival^5^. To date, the gold standard for HCC diagnosis is liver biopsy, which is an invasive procedure as well as being time-consuming and expensive. This procedure also brings difficulties and complications to the patients such as tumor seeding, intra-abdominal bleeding, pain, discomfort, bacteremia, or even death^6–8^. Therefore, non-invasive diagnostic methods are preferred nowadays. Even though detection of liver cancer in high-risk individuals has been improved by some imaging-based methods, including ultrasonography (US), computed tomography (CT), and/or high-cost magnetic resonance imaging (MRI), these radiological imaging techniques have shown some limitations like high false-positive rates leading to unnecessary patient anxiety and may need to be followed by biopsy as an invasive procedure^9^. Conventional serum markers, such as α-feto-protein (AFP), which is widely used in the clinical setting, lack specificity and show limited ability for the detection of HCC^10^. Identifying new, reliable, non or minimally invasive biomarkers is important to improve the detection of HCC.

Peripheral mononuclear cells (PBMCs) including lymphocytes and monocytes are critical components in the host immune system. Previous studies demonstrated that alteration of PBMC genes was observed in various malignancies and cancers such as non-small cell lung cancer (NSCLC), pancreatic and breast cancers^11–13^. Therefore, the detection of these changes could serve as a potential non-invasive diagnostic method. The suitable biomarker should be characterized as widely used for screening purposes and diagnosing asymptomatic patients, having high sensitivity and specificity, low cost, and easy detection.

In the present study, we integrated datasets retrieved from gene expression omnibus (GEO) and analyzed the data to identify differentially expressed genes (DEGs) between HCC, hepatitis B patients, and healthy individuals. Combining multiple datasets and integrated analysis can help in overcoming the individual dataset’s limitations, including batch effects and dataset specific biases, increasing the number of samples, generalizability, statistical power and reliability^14^.

Furthermore, enrichment and functional analyses as well as network construction were performed to find important signaling pathways and key players in HCC. Feature selection using multinomial LASSO was done to find important genes in the diagnosis of each condition. Then, multinomial logistic regression as a machine learning algorithm was used to investigate the ability of selected features to distinguish cases from controls. Gene panels with the best performance were introduced as HCC diagnostic biomarker panels and verified in a real-life patient cohort.

## Results

### Basic clinical characteristics and patients’ information

**Figure 1:**
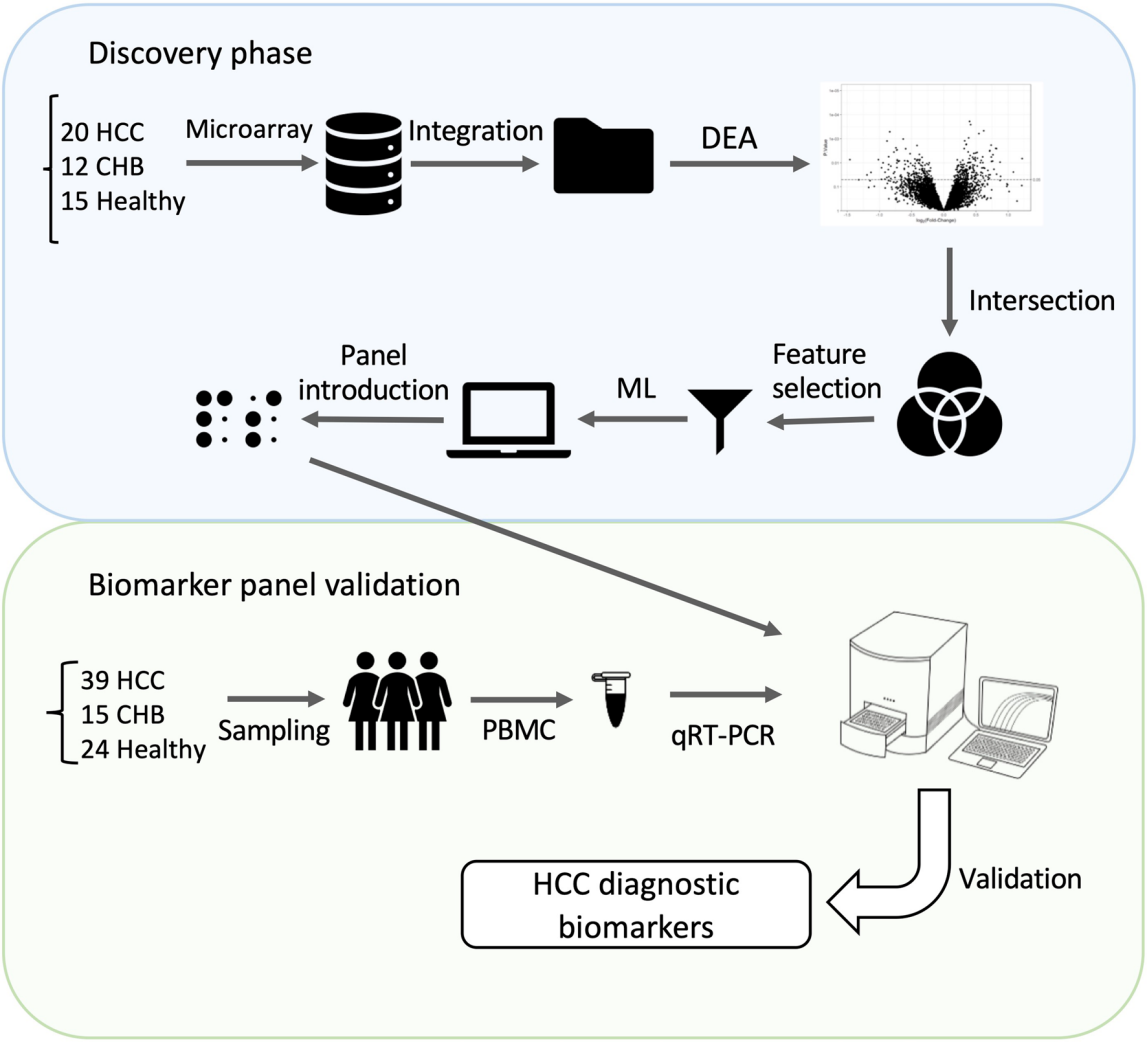
The schematic overview of the study. In discovery phase, two datasets were retrieved form GEO, integrated and analyzed to identify predictive biomarkers of HCC using feature selection and machine learning (ML) algorithm. Additionally, in the validation phase, the predictive panel was experimentally confirmed on a real-life cohort.

In this study, the gene expression profiles of HCC patients, chronic hepatitis B (CHB) patients as positive controls, and healthy individuals of the GSE49515 and GSE58208 data series were integrated to form the discovery cohort (Supplementary Fig. S1 and Supplementary Table 1). This dataset was utilized to identify significantly altered molecular mechanisms in PBMCs of HCC patients, discover potential biomarker panels and develop a machine-learning approach that can predict the diagnosis of the disease. To examine the predictive power and reproducibility, we conducted an experimental evaluation of the panel using gene expression data from 39 HCC patients, 15 CHB patients and 24 healthy controls. The study workflow is depicted in Fig. 1. For participant characteristics in the validation cohort refer to Supplementary Table 2. HCC patients comprised 29 (74.4%) men and 10 (25.6%) women. Approximately 38% of these patients reported no history of smoking, while 31% were current smoker and another 31% were former smokers. Half of the patients were overweight, and 71.8% of patients reported no family history of liver disease. Additionally, 36% of all patients reported alcohol consumption. The average total bilirubin of HCC patients was 2.51 mg/dL, while the serum albumin level averaged at 3.63 g/dL. The mean values of aspartate transaminase, alanine transaminase, and alkaline phosphatase were 58.29, 57.29, and 320.57 IU/L, respectively. Additionally, the average alpha-fetoprotein level among HCC patients was 163.7 ng/mL.

### PBMC transcriptome analysis of patients with HCC and CHB

In the present study first, we integrated samples of two datasets GSE58208 and GSE49515 by combat function of the sva package to form a unique dataset of 20 HCC, 12 CHB patients, and 15 normal controls called discovery cohort. Samples’ bar plot and PCA were plotted before and after performing combat to assure the batch effect removal (Supplementary Fig. S1).

**Figure 2:**
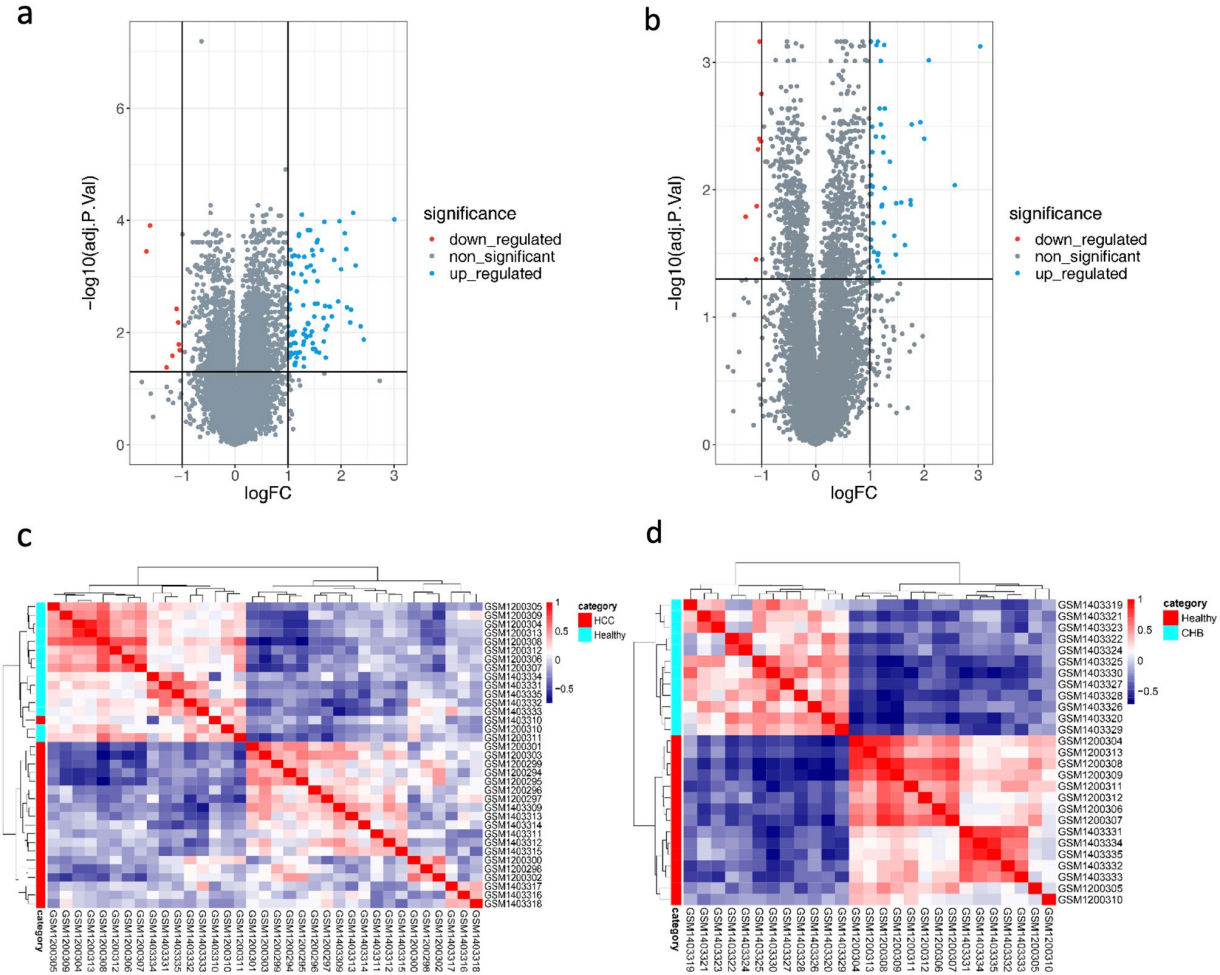
Distinct transcriptomes of HCC and CHB patients compared to healthy transcriptomes. Volcano plots show the number of statistically (adjusted *p* value < 0.05, absolute log_2_(fold change) ≥ 1) significant DEGs in PBMCs samples of (**a**) HCC and (**b**) CHB patients compared to Healthy controls. The volcano plots were created using R. Pearson correlation analysis along with the hierarchical clustering of (**c**) 107 DEGs in HCC vs. healthy and (**d**) 54 DEGs in CHB vs. healthy reveals within groups’ cohesion and altered gene expression profiles of PBMCs in HCC development. Red represents the positive correlation and blue represents the negative correlation.

**Figure 3:**
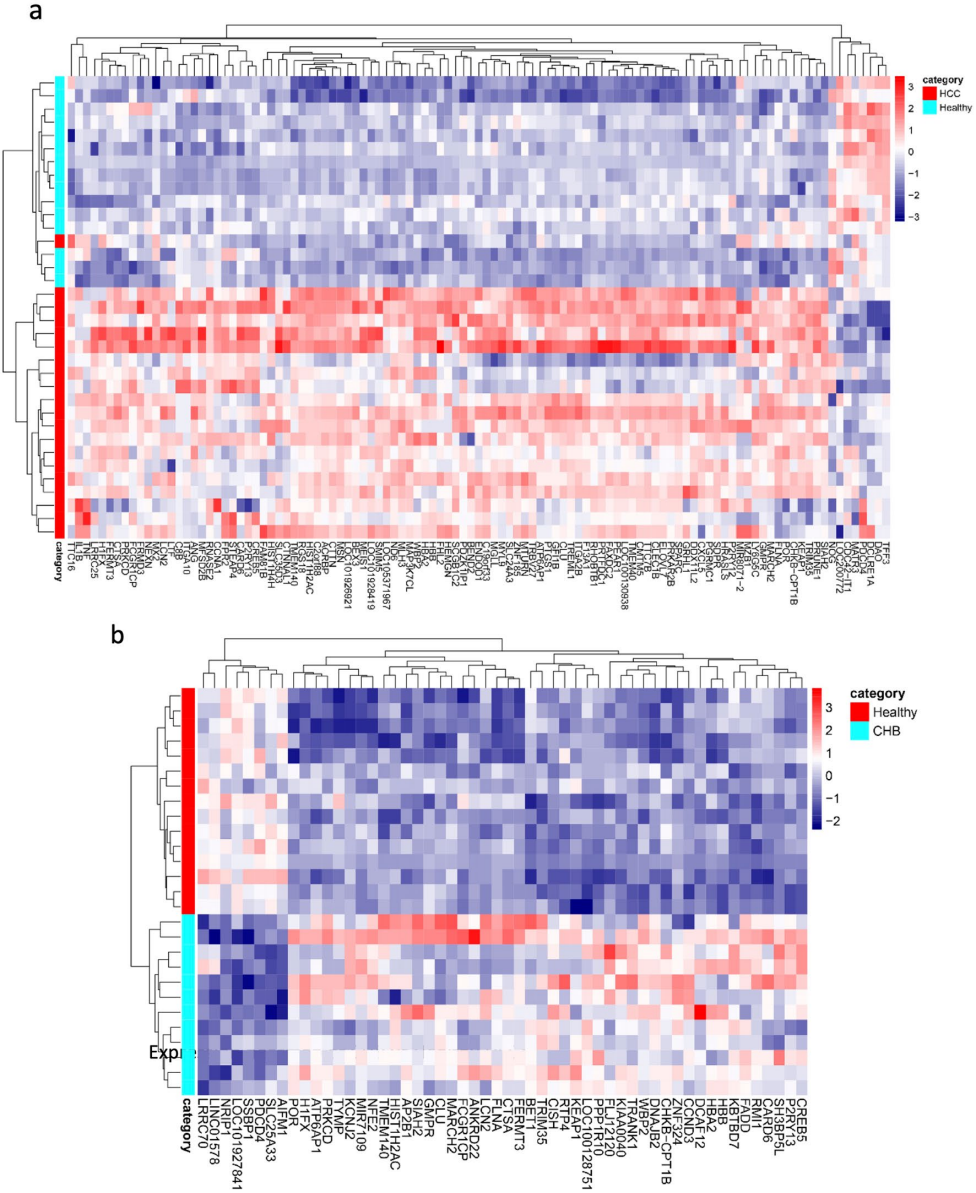
Differentially expressed genes in PBMCs that may be involved in HCC development. The expression heatmaps represent upregulated (red) and downregulated (blue) genes in the PBMCs of (**a**) HCC and (**b**) CHB patients compared to healthy controls.

A total of 107 (99 upregulated and 8 downregulated) and 54 (46 upregulated and 8 downregulated) genes were identified under the threshold of absolute log_2_(fold change) ≥ 1 and adjusted *p* value < 0.05 for HCC and CHB compared to normal conditions, respectively (Fig. 2a,b). Correlation heatmap was plotted to demonstrate the relationship between groups (Fig. 2c,d), it clearly illustrated the similarities within groups and distinction between different groups in both HCC vs healthy controls (Fig. 2c) and CHB vs healthy controls (Fig. 2d) and the conditions were greatly separated. Furthermore, we conducted hierarchical clustering and visualized the results through the heatmap. The expression patterns of 107 and 54 DEGs of HCC and CHB versus control were illustrated in Fig. 3a,b, respectively. These heatmaps demonstrated the upregulated and downregulated patterns of DEGs in each condition.

### Common and distinct gene signatures associated with HCC in PBMC

**Figure 4:**
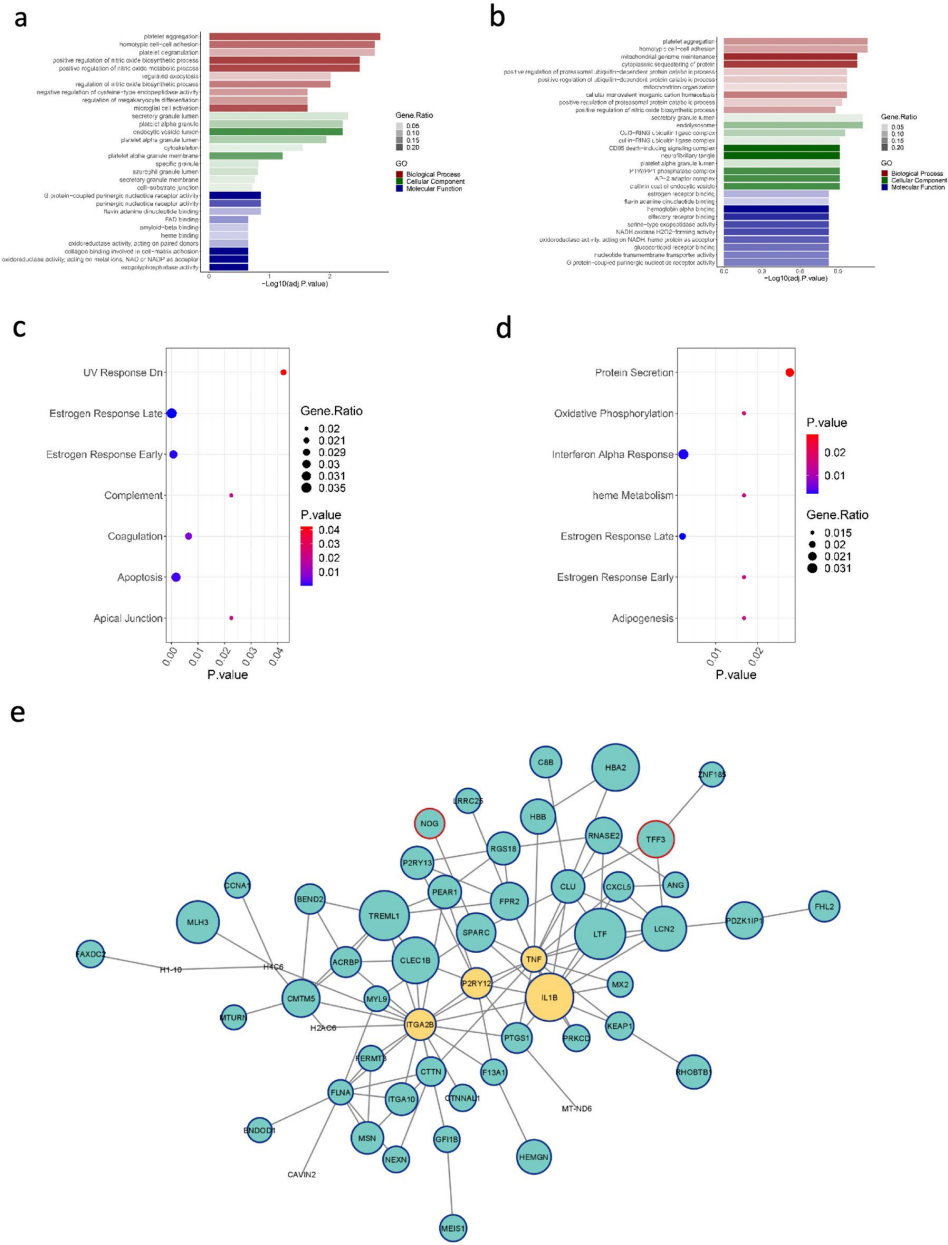
Gene ontology, signaling pathway and network analyses in HCC. Top 10 enriched biological processes, cellular components and molecular functions in (**a**) HCC and (**b**) CHB compared to healthy individuals. The most significant pathways of (**c**) HCC vs. healthy controls and (**d**) CHB vs. healthy controls. (**e**) PPI network of DEGs in HCC was created using Cytoscape (3.9.1). The network consists of 96 nodes and 107 edges. The size of the nodes corresponds to absolute log_2_(fold change). Blue and red border lines indicate the upregulated and downregulated genes, respectively. Yellow balls represent the hub genes of the network based on the betweenness, closeness, degree, eccentricity, radiality, and stress centralities.

We next investigated the gene ontologies (GOs) of DEGs in HCC (Fig. 4a) and CHB (Fig. 4b) patients compared to healthy controls using Enrichr. Terms and processes with *p* value < 0.05 were considered significant and the top 10 biological processes (BP), molecular functions (MF), and cellular components (CC) were visualized for each condition in comparison to normal controls. Platelet aggregation was the most enriched BP when comparing both HCC and CHB groups to healthy subjects. The secretory granule lumen was among enriched CC in both comparisons. Furthermore, G-protein-coupled purinergic nucleotide receptor activity and hemoglobin alpha binding were enriched MF in HCC and CHB comparisons with the healthy group, respectively. Pathway analysis demonstrated that “estrogen response” was involved in both HCC and CHB pathogenesis (Fig. 4c,d). Furthermore, enrichment analysis showed that DEGs in the HCC group were significantly members of “apoptosis” and “coagulation” signaling pathways (Fig. 4c), while “Interferon alpha response” was a substantially enriched pathway in CHB patients compared to normal controls (Fig. 4d).

### The protein–protein interaction network of DEGs in HCC

Significant altered genes of HCC compared to the healthy group were applied to construct the PPI network using STRING. Visualization of the network was achieved using Cytoscape, highlighting functional interactions. The network comprised 96 nodes and 107 edges as shown in Fig. 4e. Centrality parameters, including betweenness, closeness, degree, eccentricity, radiality, and stress of the nodes, were computed with the CentiScape plugin. The top 10 nodes with the highest values for each centrality were identified. Subsequently, genes appearing in the top 10 across all centrality measures were recognized as hub genes. Key nodes, according to the CentiScaPe analysis, included tumor necrosis factor (TNF), interleukin 1 beta (IL-1β), integrin subunit alpha 2b (ITGA2B), and purinergic receptor P2Y12 (P2RY12), as detailed in Supplementary Fig. S2.

### Discovery of candidate predictive biomarker panels in PBMCs of HCC patients

**Figure 5:**
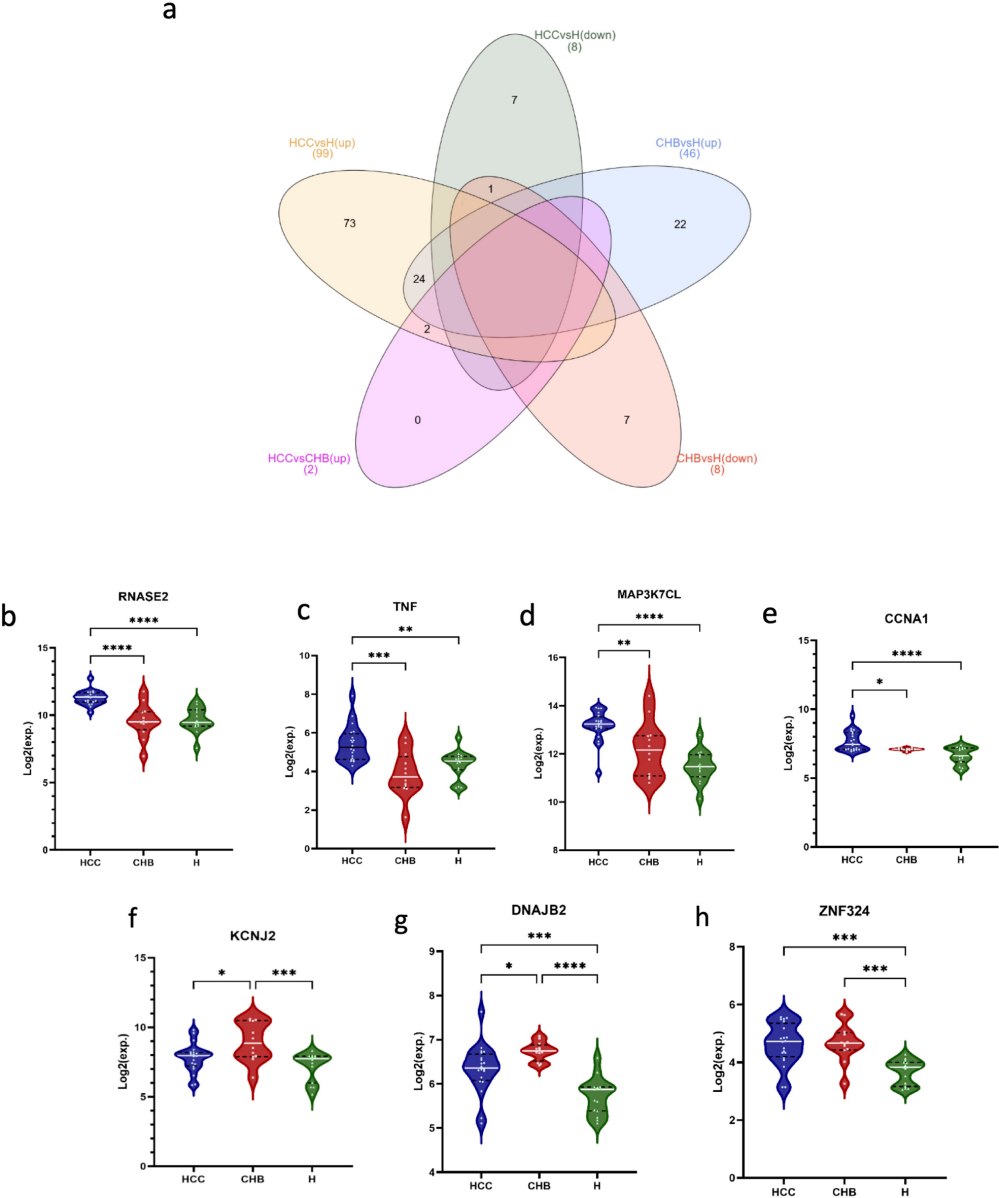
Identification of specific genes for HCC and CHB cases. (**a**) Venn diagram represents the intersection between upregulated and downregulated genes in HCC and CHB compared to healthy controls. It demonstrated that there are 73, and 22 unique upregulated genes for HCC and CHB compared to healthy controls, respectively. The violin plots represent the expression levels of (**b**) RNASE2, (**c**) TNF, (**d**) MAP3K7, (**e**) CCNA1, (**f**) KCNJ2, (**g**) DNAJB2, and (**h**) ZNF324. X-axis represents HCC (left), CHB (middle) and healthy control (right), Y-axis represents mRNA expression in log2.

To investigate the intersection of DEGs, a Venn diagram was constructed using all significant up regulated and down regulated genes in HCC and CHB compared to healthy controls (Fig. 5a). Upregulated genes that did not have an overlap for each condition compared to healthy controls were then selected for the feature selection step. There were 73 unique genes that were only upregulated in HCC compared to healthy controls, while 22 genes were upregulated in CHB in comparison with healthy controls. Furthermore, there were two genes that not only upregulated in HCC compared to normal samples but also upregulated in HCC patients compared to CHB ones. These 97 genes were then fed to multinomial LASSO for feature selection in R. A list of 11, 7, and 5 genes with non-zero LASSO coefficient were reported in Supplementary Table 3, which that may have the potential to detect HCC, CHB, and healthy, respectively.

To assess the diagnostic performance of candidate genes in distinguishing the three specified groups, we utilized the top three genes with the highest absolute LASSO coefficient for each condition. These genes included CCNA1, ANG, RNASE2, FADD, TNF-α, KCNJ2, ZNF324, DNAJB2, and MAP3K7, allowing for the creation of 3- to 7-biomarker panels. Then we used multinomial logistic regression, and built a model for each 3- to 7-marker panel that is evaluated by fivefold cross-validation. Sensitivity and specificity of the best panels for each condition were reported in Table 1. The best 3-marker panel, comprising RNASE2, TNF-α, and DNAJB2 genes, demonstrated an accuracy of 0.915. The accuracy increased with the addition of more markers: 0.936, 0.957, and 0.979 for the 4-, 5-, and 6-marker panels, respectively. It reached 1 for the 7-marker panel, which consists of RNASE2, TNF-α, KCNJ2, CCNA1, FADD, ZNF325, and MAP3K7 genes. The expression profiles of these genes, contributing to the construction of the best 3- to 7-marker panels, are illustrated in Fig. 5b–h. The results indicated significant upregulation of RNASE2, TNF-α, CCNA1, and MAP3K7 in HCC compared to both CHB and healthy controls. Additionally, DNAJB2 and KCNJ2 were found to be upregulated in CHB relative to both HCC and healthy controls. ZNF324, on the other hand, was upregulated in both HCC and CHB when compared to healthy controls.

**Table 1:** The best 3- to 7-marker panels on microarray data (discovery cohort).

Panel	No. features	HCC sen	CHB sen	Healthy sen	HCC spe	CHB spe	Healthy spe	Accuracy
RNASE2, TNF-α, DNAJB2	3	0.95	0.833	0.933	0.963	0.943	0.969	0.915
RNASE2, TNF-α, MAP3K7, KCN2	4	0.95	0.917	0.933	0.926	1	0.969	0.936
RNASE2, TNF-α, MAP3K7, KCN2, DNAJB2	5	0.95	0.917	1	0.963	0.971	1	0.957
RNASE2, TNF-α, KCNJ2, CCNA1, FADD, ZNF324	6	1	0.917	1	1	1	0.969	0.979
RNASE2, TNF-α, KCNJ2, CCNA1, FADD, ZNF325, MAP3K7	7	1	1	1	1	1	1	1

### Experimental validation of the best *in-silico* predictive panels in the real-life HCC patient cohort

**Figure 6:**
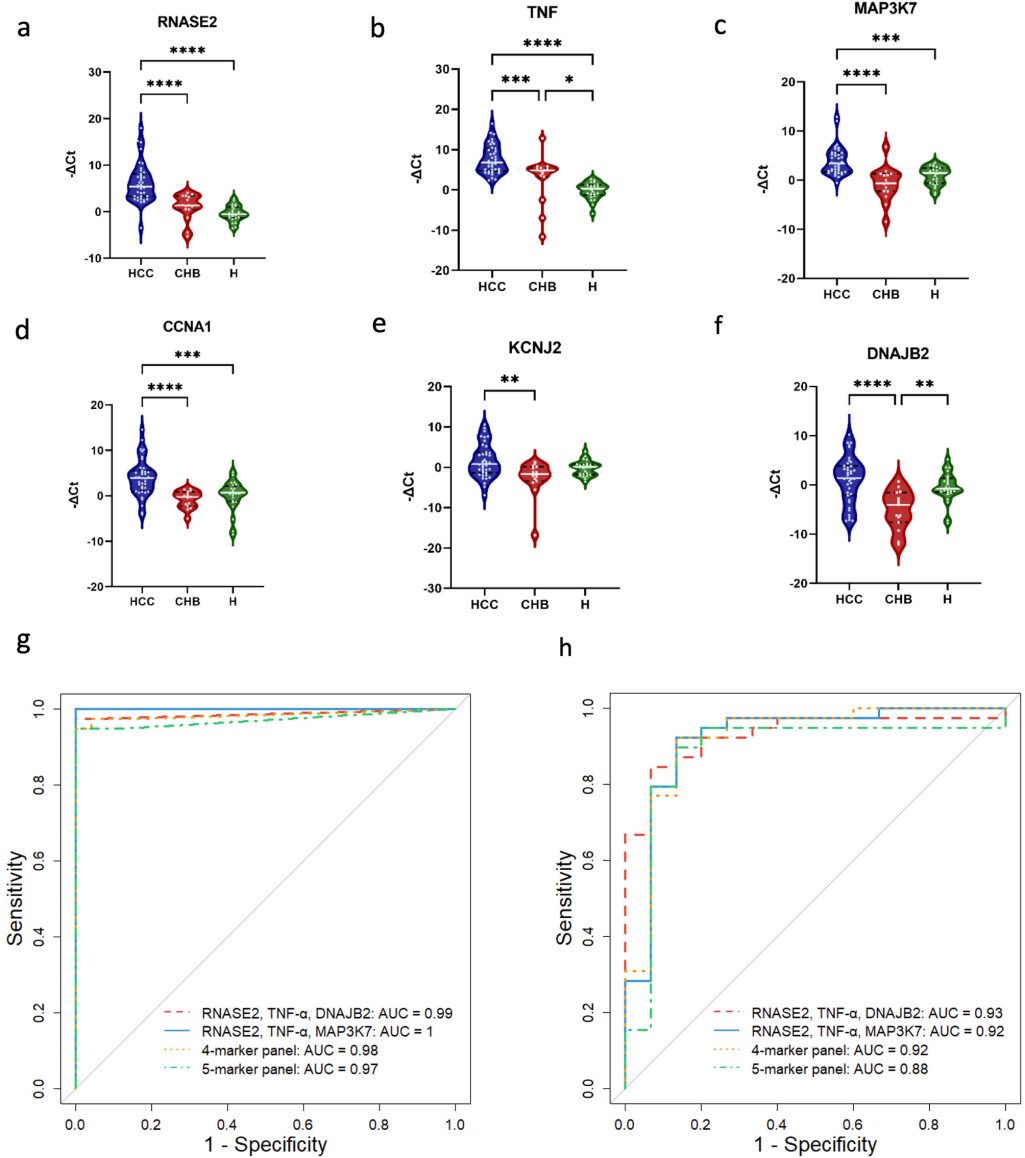
Real-life cohort evaluation of predictive panels and ROC curves for the 3- to 5-marker panels. Experimental validation of the predictive panels in the real-life cohort. Relative expression of candidate markers (**a**) RNASE2, (**b**) TNF, (**c**) MAP3K7, (**d**) CCNA1, (**e**) KCNJ2, and (**f**) DNAJB2 in PBMC samples of patients with HCC, CHB and healthy controls. Green violin plots represent the expression levels of genes in normal patients (n = 24), blue and red violin plots represent the expression levels of genes in patients with HCC (n = 39) and CHB (n = 15), respectively. Relative expression of genes represents as -ΔCt on y-axis. (**g**) The ROC curve depicts the performance of the three-marker panel comprising RNASE2, TNF-α, and DNAJB2 (red line), as well as the four-marker (yellow line) and five-marker (green line) panels, alongside the best introduced three-marker panel containing RNASE2, TNF-α, and MAP3K7 (blue line) for distinguishing HCC from healthy controls. (**h**) The ROC curve illustrates the ability to distinguish HCC from CHB, showing the performance of the three-marker panel containing RNASE2, TNF-α, and DNAJB2 (red line), the best three-marker panel introduced, RNASE2, TNF-α, and MAP3K7 (blue line), as well as the four-marker (yellow line) and five-marker (green line) panels. The AUC is displayed in the plot.

The expression of gene members in top models were then evaluated using qRT-PCR. Their expression levels were plotted using GraphPad Prism version 9. It was observed that RNASE2, TNF-α, MAP3K7, and CCNA1 were significantly differentially expressed in HCC patients compared to healthy controls. As expected, DNAJB2 and KCNJ2, showed no significant changes in HCC compared to healthy samples (Fig. 6a–f). Furthermore, RNASE2, a gene that was upregulated in HCC compared to CHB in the discovery cohort, also exhibited a similar upregulation pattern in HCC compared to CHB in the experimental results.

Sensitivity, specificity, accuracy and AUC of these models were described in Table 2. The best in silico 3-marker panel achieved an accuracy of 0.984 and an AUC of 0.99 based on experimental data. The 3-marker panel demonstrated the highest performance (Fig. 6g), suggesting that increasing the number of markers can lead to increased complexity of the model and decreased generalization ability.

**Table 2:** Performance of machine learning on the real-life patient cohort data (validation cohort).

Panel	Sensitivity	Specificity	Accuracy	AUC
RNASE2, TNF-α, DNAJB2	0.974	1	0.984	0.99
RNASE2, TNF-α, MAP3K7, KCNJ2	0.949	1	0.968	0.98
RNASE2, TNF-α, MAP3K7, KCNJ3, DNAJB2	0.949	1	0.968	0.97

Furthermore, we conducted machine learning on other combinations of 3-marker panels with the biomarkers presented in the best 4-marker panel. The panels consisting of RNASE2, TNF-α, and MAP3K7 demonstrated the best performance with an AUC of 1 (Supplementary Table 4).

Additionally, the best 3- to 5-marker panels, along with the best 3-marker panel from assessing the combinations, were utilized to build models to detect HCC from CHB. Results indicated that the 3-marker panel comprising RNASE2, TNF-α, and MAP3K7, which showed the best performance in detecting HCC from healthy controls, also exhibited acceptable performance in distinguishing HCC from CHB patients with an accuracy of 0.907 and AUC of 0.92 (Supplementary Table 5).

## Discussion

HCC is the most common type of primary liver cancer with a raising global burden of mortality. Being asymptomatic in the early stages delays timely diagnosis and leads to limited curative options in advanced stages^15^. PBMCs including monocytes, lymphocytes, and natural killer (NK) cells have a critical role in immune system functions. Studies showed that they have applications in the diagnosis, screening, and prognosis of diseases. Accumulating evidence has indicated that gene expression and methylation profiles of PBMCs alter in different types of disorders and malignancies^16^, such as HCC^17^. Alpha-fetoprotein (AFP) and imagining techniques are conventional methods for screening and early detection of HCC. However, the sensitivity of these methods even in combination is still suboptimal, ranging from 39 to 65% for AFP^18^ and just 63% for the combination of ultrasound and AFP^19^, highlighting the need for more reliable and accurate methods to improve the prognosis and diagnosis of HCC patients. Furthermore, transcriptome analysis and identifying key players could help to understand the underlying mechanism of tumorigenesis of HCC^20^.

In this study, two different datasets, retrieved from the GEO database, were integrated to improve the sample size and reduce the study bias. The unified dataset which contained 20 HCC, 12 CHB patients, and 15 healthy controls was used to identify genes with significant alteration in their expression known as DEGs. Our findings showed that 107 and 54 genes were significantly expressed in HCC and CHB compared to healthy controls, respectively. Functional annotation analysis that was conducted using DEGs, revealed that platelet aggregation was the common enriched term among the BPs for both HCC and CHB conditions in comparison to healthy controls. Accumulating evidence suggests that platelets play a pivotal role in both inflammation and the progression of cancer^21,22^. Their contact with PBMCs modulated the immune response against viral infections^23^. Furthermore, it has been observed that platelets modulate T cell immunity against cancer, emphasizing they role in the immune response in the tumor microenvironment^24^. The interaction of platelet-leukocyte, as a predominant member of PBMCs, can lead to inflammatory immune reactions in different diseases^25^, including cancer^26^. The effector functions of innate leukocyte are modulated by platelets that are involved in immune responses but also promote thrombosis and metastasis^26^.

Platelets are small anucleate cellular fragments, that has functional organelles including mitochondria, Golgi apparatus, and endoplasmic reticulum (ER). Megakaryocytes generate platelets in bone marrow and release them into the blood stream^27–29^. In homeostasis and the initiation of coagulation, platelets play a crucial role by binding to injured vascular sites, forming aggregates, and subsequently arresting bleeding^28,29^. The role of platelets extends beyond clotting. Notably, they possess mRNA, which equips them with the capability to synthesize, express, and release proteins^28,29^. Upon activation, platelets initiate the release of various bioactive molecules, including multiple growth factors, serotonin, inflammatory cytokines, tumor necrosis factor alpha, chemokines, adrenaline, and more, via platelet granules. Bioactive molecules released from activated platelets can exhibit multifaceted effects, such as shielding cancer cells against immune surveillance, creating a stimulant environment for tumor growth, invasion and facilitating metastasis, or even exerting all of these actions concurrently^22,29,30^. While a reduction in platelet counts has been consistently reported in patients afflicted with chronic HBV infection and various other chronic liver diseases, it is noteworthy that an elevated platelet count has frequently been associated with HCC aggressiveness and tumor size, an increased propensity for tumor recurrence, and an elevated risk of metastatic dissemination^30^. Henceforth, conventional antiplatelet medications or platelet activation inhibitors may have the capacity to impede platelet aggregation, diminish hepatocyte damage arising from viral infection, and suppress the development of HCC^30^. Moreover, multiple BPs related to platelet function demonstrated significant enrichment in HCC compared to healthy controls. Key processes included platelet degranulation, which pertains to the platelet response to aggregating agents, and the regulation of megakaryocyte differentiation.

Other significant BPs in HCC against healthy controls were regulation of nitric oxide synthetic and metabolic processes. Nitric oxide (NO) serves as a transcellular signaling molecule, and its production is catalyzed by enzymes known as nitric oxide synthases (NOS). All isoforms of NOS have been identified in tumor cells, and they play a pivotal role in both promoting and suppressing cancer progression. NOS exhibits a dual role in cancer pathogenesis, correlating with tumor grade, proliferation rate, and the expression of cancer-development signaling molecules, such as the estrogen receptor. As a free radical gas molecule at room and body temperature, NO is highly diffusible and participates actively in various BPs due to its high reactivity. It generates metabolites that are central to genotoxic effects, including DNA damage. High concentrations of NO can hinder cancer growth and trigger cell apoptosis, whereas lower concentrations can stimulate tumor growth and proliferation^31^.

Furthermore, there were several remarkable pathways in HCC such as estrogen response, that known to be involved in the development and progression of cancers^32,33^, including HCC^34^. Coagulation was another significantly enriched pathway in HCC vs healthy controls. Various studies indicated the role of blood coagulation proteins in tumor progression^35^. Since the main site of coagulation factor production is liver, it has been suggested that these factors could be associated with the progression of HCC^36^.

In this study, we constructed a biological network based on the detected DEGs. The genes TNF-α, IL1B, ITGA2B, and P2RY12 were subsequently identified as hub genes in the PBMCs of HCC and can be considered therapeutic targets in HCC management. TNF-α is a crucial cytokine implicated in numerous signaling pathways, including inflammation, immunity, and even tumorigenesis. Predominantly generated by macrophages, its production is also observed in other cell types such as endothelial cells^37^. Its overexpression has been observed in monocytes isolated from PBMCs of colorectal cancer patients in comparison to normal controls^38^. However, in the liver, TNF-α possesses a dichotomous role: it can instigate liver tissue damage through hepatocyte apoptosis, while concurrently stimulating a regenerative effect and possibly promoting hepatocarcinogenesis via cell proliferation. Experimental findings suggested that complete inhibition or ablation of TNF-α attenuates liver cancer progression in laboratory mice. However, this modulation also correlates with reduced survival and the onset of hepatic failure.

Dysregulation of IL-1, another hub gene in the network, has been reported to be associated with various types of human malignancies. Accumulating evidence suggests that IL-1 plays an important role in tumorigenesis, cancer progression, metastasis, and the response to cancer treatment. IL-1β is one of the main agonists of the IL-1 family^39^ which is produced by tumor and immune cells^40^. The serum level of IL-1B shows an elevation in the case of hepatitis, cirrhosis, liver fibrosis and HCC. The elevation of IL-1β serum levels can lead to overexpression of gankyrin which is an oncoprotein. Its overexpression induces the cell growth, invasiveness, and metastasis by regulating IL-1β/IRAK-1 signaling^39^. COX-2 expression that is also induced by IL-1β, inhibits antigen presenting cells from maturation and activation at the tumor microenvironment^40^.

ITGA2 constitutes one of the pivotal subunits, forming a heterodimer along with the β1 subunit. The protein encoded by this gene functions as a transmembrane receptor integral to cellular adhesion processes, facilitating both cell–cell adhesion and the adherence of platelets and various cell types to the extracellular matrix. Moreover, it involves in cell surface-mediated signal transduction, modulating cellular growth and differentiation through the interaction with growth factors and chemokines^41^. In normal tissues and organs, the expression of ITGA2 remains very low; however, its overexpression has been identified in multiple cancer types, including HCC^42^. This is attributed to its role in critical cellular processes such as tumor cell proliferation, migration, invasion, angiogenesis, and metastasis^41,43^.

P2RY12 is an adenosine diphosphate (ADP)-responsive G protein–coupled receptor, whose expression is not exclusive to the surface of platelets but is also prominently observed in lymphocytes, monocytes, and megakaryocytes^44^, as well as cancer cells^45^. Research has elucidated that adenosine triphosphate (ATP) and ADP are secreted into the tumor site by both platelets and tumor cells, serving as activators for P2Y12, subsequently modulating the inflammatory response. Further investigations have shown that hepatic macrophages also express P2Y12 receptor. This receptor plays a crucial role in the induction of ER stress pathways. The induction of these pathways has a pivotal role in the pathogenesis of chronic liver diseases and HCC^46^.

In the next step, a computational feature selection was conducted on the selected DEGs. LASSO algorithm was used for multinomial computational feature selection. The three most significant features with the most absolute coefficient in each condition were utilized to construct 3 to 7-biomarker panels. Panels with the best performances included TNF-α, RNASE2, MAP3K7, CCNA1, DNAJB2, and KCNJ2.

Ribonuclease A family member 2 or RNASE2, also known as eosinophil-derived neurotoxin (EDN), belongs to the RNase superfamily, playing a pivotal role in the immune system and host defense mechanisms against pathogens. Upon activation by proinflammatory stimuli, eosinophils secrete a suite of proteins, with EDN being one of the four major proteins^47^. Additionally, monocyte-derived macrophages produce EDN upon stimulation, demonstrating capabilities in inducing cell proliferation, migration, and invasion^48^. Recent literature reports an overexpression of EDN in various tumors^48–50^. While existing studies have underscored the influence of immune-related genes on the tumor microenvironment, facilitating the progression of HCC^51,52^, this study marks the inaugural mention of RNASE2 in the context of HCC.

Mitogen-activated protein kinase kinase kinase 7 or MAP3K7, which is also known as transforming growth factor-β activated kinase 1 (TAK1), is a pivotal member of the MAPKKK family. This kinase can be activated by a plethora of molecules, encompassing TGF-β, cytokines (e.g., TNF-α and IL-1), Toll-like receptors, CD40, and B cell receptors. Additionally, several viruses, notably HBV and HCV, which are associated with a heightened risk of HCC, can induce MAP3K7 activation. Notably, there exists a positive correlation between MAP3K7 and the expression of the mammalian target of rapamycin (mTOR), which is often linked to poor survival in HCC patients^53^. Furthermore, MAP3K7 can activate pathways such as NF-κB and MAPK, both of which are implicated in tumorigenesis^54^. Consequently, in agreement with our findings, overexpression of MAP3K7 can play a pivotal role in the inflammation and the progression of HCC^53^.

Among biomarker candidates, Cyclin A1 is a member of the A-type cyclin protein family that plays a pivotal role in regulating the G1/S phase transition of the cell division cycle. This regulation is achieved through its interaction with cyclin-dependent kinase 2 (CDK2) and cell division cycle 2 (CDC2) kinase. Additionally, Cyclin A1 has been identified to be associated with key cell cycle regulators such as retinoblastoma (Rb), p21 family proteins, and transcription factor E2F-1^55^. Dysregulation of Cyclin A1 has been implicated in various cancers including breast and thyroid cancers^56^. While the roles of cyclins CCNE1 and CCNB1 in HCC have been elucidated^57,58^, the involvement of CCNA1 in HCC remains unreported.

Potassium inwardly rectifying channel subfamily J member 2 or KCNJ2 belongs to the potassium inwardly rectifying channel family. Previous studies have highlighted the oncogenic roles of members of this family in various cancers. For instance, the interaction between KCNJ2 and the HIF1α transcription factor has been shown to establish a positive-feedback loop, contributing to osteosarcoma metastasis^59^. Furthermore, inhibition of KCNJ2 has been associated with reduced proliferation, migration, and epithelial-mesenchymal transition (EMT) progression in papillary thyroid carcinoma cells^60^. In prostate cancer cells, KCNJ2 promotes proliferation by binding to the RELA protein in the nucleus, thereby activating the NF-κB signaling pathway^61^. Based on these findings, it is suggested that KCNJ2 might exert similar effects on HCC cells.

DnaJ heat shock protein family (HSP40) member B2, a member of HCC biomarker panels, belongs to HSP40 subclass DNAJB proteins. Emerging evidence indicates that DNAJB proteins play a crucial role in cancer invasion and metastasis by modulating diverse signaling pathways. However, certain members, such as DNAJB2, remain under-investigated. Even with limited data on these specific DNAJB proteins, they are believed to have a potential role in cancer development^62^.

While previous studies have been conducted to identify biomarkers for diagnosing HCC^63^, some have merely identified and introduced biomarkers based on bioinformatic analyses, lacking validation in independent real-life patient cohorts^64,65^. Using feature selection method, 3- to 7-biomarker panel was utilized as machine learning entry. Then, multinomial logistic regression algorithm was performed with fivefold cross validation to prevent the model from overfitting. Performing machine learning on experimental data obtained from an independent patient cohort and finding a 3-marker panel with the best performance confirmed the *in-silico* results.

Although some studies have introduced diagnostic biomarkers for HCC in PBMCs^8,66^, we utilized an integrated dataset comprising two distinct datasets. This approach was employed to eliminate batch effects from individual studies and to build a generalized predictive model using multi-centric data. Furthermore, this study represents a novel application of a predictive machine learning approach in PBMC samples for HCC, focusing on creating reliable models and accurately predicting this cancer from healthy controls and CHB that shares similar signaling pathways and biological processes with HCC. However, the performance of the introduced panels should be further investigated in a larger cohort to ensure their applicability in a clinical setting.

In conclusion, DEGs were identified from PBMC microarray data of HCC and CHB patients in the present study. Upregulated DEGs for each condition were then fed to feature selection method and a combination of features was determined to develop an input for machine learning. The best model of each 3-,4-, and 5-biomarker panels were confirmed by qRT-PCR, and the 3-marker panel of TNF-α, RNASE2, and MAP3K7 found to have the best performance on experimental data, suggesting that it has the potential to be used as a non-invasive diagnostic panel with high accuracy for detecting HCC compared to healthy controls and CHB patients.

## Material and methods

### Data collection and sample preparation

The GEO database (https://www.ncbi.nlm.nih.gov/geo/) was searched and two gene expression profile datasets (GSE49515 and GSE58208) that met our criteria were downloaded. Both of these datasets were based on the GLP570 platform (Affymetrix Human Genome U133 Plus 2.0 Array); therefore, probe annotation files were utilized for further analysis. One of the dataset profiles (GSE49515) consisted of 10 PBMC samples of HCC patients and 10 normal controls and the other one (GSE58208) contained the data of 10 HCC, 5 healthy controls, and 12 CHB samples as positive controls. These two datasets were then integrated and utilized as the discovery cohort.

Additionally, blood samples were obtained in EDTA tube from 39 patients with HCC and 15 CHB, who were diagnosed at Taleghani Hospital, Shahid Beheshti University of Medical Sciences (SBMU), and 24 healthy controls, between 2021 and 2023. The demographic characteristics of patients were also collected. These features included age, gender, family history of liver disease, overweight, drinking, smoking state, total bilirubin, serum albumin, aspartate transaminase, alanine transaminase, alkaline phosphatase, platelet count, and alpha fetoprotein. The blood samples of healthy controls were also collected from individuals without any evidence of liver diseases or HCC. Written informed consent was obtained from each participant and/or their legal guardian(s) and this research was approved by the ethical review board of Shahid Beheshti University of Medical Sciences, Tehran, Iran (IR.SBMU.RIGLD.REC.1402.0200). All methods were carried out in accordance with relevant guidelines and regulations. PBMC isolation was permed using Ficoll density gradient sedimentation (Lymphodex, Inno-Train, Germany).

### RNA isolation and quantitative reverse-transcription PCR

RNA isolation was performed on PBMCs using the TRIzol (Qiagen, USA) reagent based on the manufacturer’s instruction. Then, cDNA was synthesized using cDNA synthesis kit (Parstous, Iran) in the presence of random hexamers. The synthesized cDNAs were stored at − 20 °C for further experiments. Expression levels of the genes were quantified by qRT-PCR. Briefly, quantitative real-time PCR (qRT-PCR) reactions were performed on a Rotor Gene Q System (QIAGEN) using SYBR Green master mix (AmpliQon, Denmark). The ΔCt was calculated for the obtained data. GAPDH (Glyceraldehyde- 3-phosphate dehydrogenase) was used as an internal control gene. The detailed information about the primers is presented in Supplementary Table 6.

### Identification of DEGs

First, we utilized the combat function from the sva package in R to integrate datasets, improve the number of samples, and remove the batch effect. Then we performed differential analysis to statistically compare HCC, CHB, and normal samples. The genes with absolute log_2_(fold change) ≥ 1 and adjusted *p* value < 0.05 in all comparisons including HCC vs. healthy, CHB vs. healthy, and HCC vs. CHB were considered as statistically differentially expressed. Limma package was used to determine DEGs in R. The correlation heatmap was plotted to illustrate the relationship between groups using pearson correlation and the expression heatmap was used to explore the relationship of DEGs.

### Enrichment and functional analysis

We utilized Enrichr online tool (https://maayanlab.cloud/Enrichr/) to detect GO categories, including biological process (BP), molecular function (MF), and cellular components (CC) as well as signaling pathway analysis. Results with *p* < 0.05 were considered significant. The results of top GOs and pathways were visualized using R.

### Biological network construction

DEGs were uploaded into the Search Tool for Retrieval of Interacting Genes/Proteins (STRING) (https://string-db.org/), which is an online tool designed for predicting protein–protein interactions (PPI), to find out the functional interactions between the proteins encoded by identified DEGs and construct a PPI network. For this purpose, all DEGs of HCC compared to healthy controls were uploaded to the STRING database. Various types of evidence, including text mining, experiments, databases, neighborhood, gene fusion, co-occurrence, and co-expression, were selected as sources of interaction. The minimum required interaction score was set at 0.4, meaning that only interactions above this threshold were included in the predicted network. The network was visualized using Cytoscape (version 3.9.1). The CentiScape plugin was employed to examine various parameters of the nodes, which included betweenness, closeness, degree, eccentricity, radiality, and stress. Furthermore, enrichment analysis was conducted to investigate BPs, MFs, and CCs associated with the network.

### Feature selection and machine learning

First, an online tool was used to obtain a Venn diagram of DEGs for each condition and healthy controls. Then, to identify the most important and effective genes among DEGs, unique upregulated DEGs selected from the Venn diagram were fed to the multinomial LASSO algorithm in R. Top 3 features in each condition (HCC, CHB, and healthy controls) were used to create a 3- to 7-marker panels of gene combinations. Multinomial logistic regression was used as a machine learning algorithm to perform classification and determine the diagnostic value of each model using glm in R. Since the sample size was small, logistic regression would be an appropriate choice for the classification. Five-fold cross-validation was used to evaluate each model and evaluation parameters such as sensitivity, specificity and accuracy was reported for each condition. pROC package was also utilized to plot the receiver operating characteristic (ROC) curves and estimate the area under the ROC curve (AUC).

To validate the potential predictive ability of the panels, a machine learning approach was employed using a real-life cohort consisting of 39 HCC, 15 CHB patients and 24 healthy individuals. For this step, binomial logistic regression was used. In addition to being suitable for a small number of samples, as mentioned previously, this algorithm is appropriate for building models for disease state (diseased/healthy) classification and decision-making problems and has been widely employed in health sciences studies. Therefore, this algorithm was used to build models based on the expression results obtained from qRT-PCR. Five-fold cross-validation was employed to improve the reliability of the model and to prevent overfitting.

### Statistical analysis

We performed statistical analysis using GraphPad Prism version 9. The comparison of selected genes between conditions in the discovery cohort was analyzed using one-way ANOVA, while t-test was employed in the validation cohort. p-value < 0.05 was considered a statistically significant difference.

### Supplementary Information

Supplementary Information.

## Data Availability

The data generated during the current study is available from the corresponding author on reasonable request.
