# Supplementary material for: Predictive three-biomarker panel in peripheral blood mononuclear cells for detecting hepatocellular carcinoma
Source: Sci Rep. 2024 Mar 29;14:7527. doi: 10.1038/s41598-024-58158-9 (PMC10980807; doi:10.1038/s41598-024-58158-9)
Supplement: Supplementary file 1 — Supplementary Information. [file 41598_2024_58158_MOESM1_ESM.pdf]

Predictive three-biomarker panel in peripheral blood mononuclear cells for detecting hepatocellular carcinoma

Sara Fayazzadeh[1], Mahsa Ghorbaninejad[2], Amirhassan Rabbani[3], Javad Zahiri[1,4], Anna Meyfour[2*]

1. Bioinformatics and Computational Omics Lab (BioCOOL), Department of Biophysics, Faculty of Biological Sciences, Tarbiat Modares University, Tehran, Iran
2. Basic and Molecular Epidemiology of Gastrointestinal Disorders Research Center, Research Institute for Gastroenterology and Liver Diseases, Shahid Beheshti University of Medical Sciences, Tehran, Iran
3. Department of Transplant & Hepatobiliary Surgery, Taleghani Hospital, Shahid Beheshti University of Medical Sciences, Tehran, Iran
4. Department of Neurosciences, University of California, San Diego, CA, USA

*Corresponding author

Anna Meyfour, Research Institute for Gastroenterology and Liver Diseases, Shahid Beheshti University of Medical Sciences, Arabi Ave., Daneshjoo Blvd., Velenjak, Tehran, Iran. Postal Code: 1985717413, Tel: +98 21 22432521. Email: a.meyfour@sbmu.ac.ir

Supplementary Figure 1. PCA analysis a) before and b) after batch effect removal. Distribution of expression values in samples of GSE49515 and GSE58208 studies c) before and d) after batch effect correction.

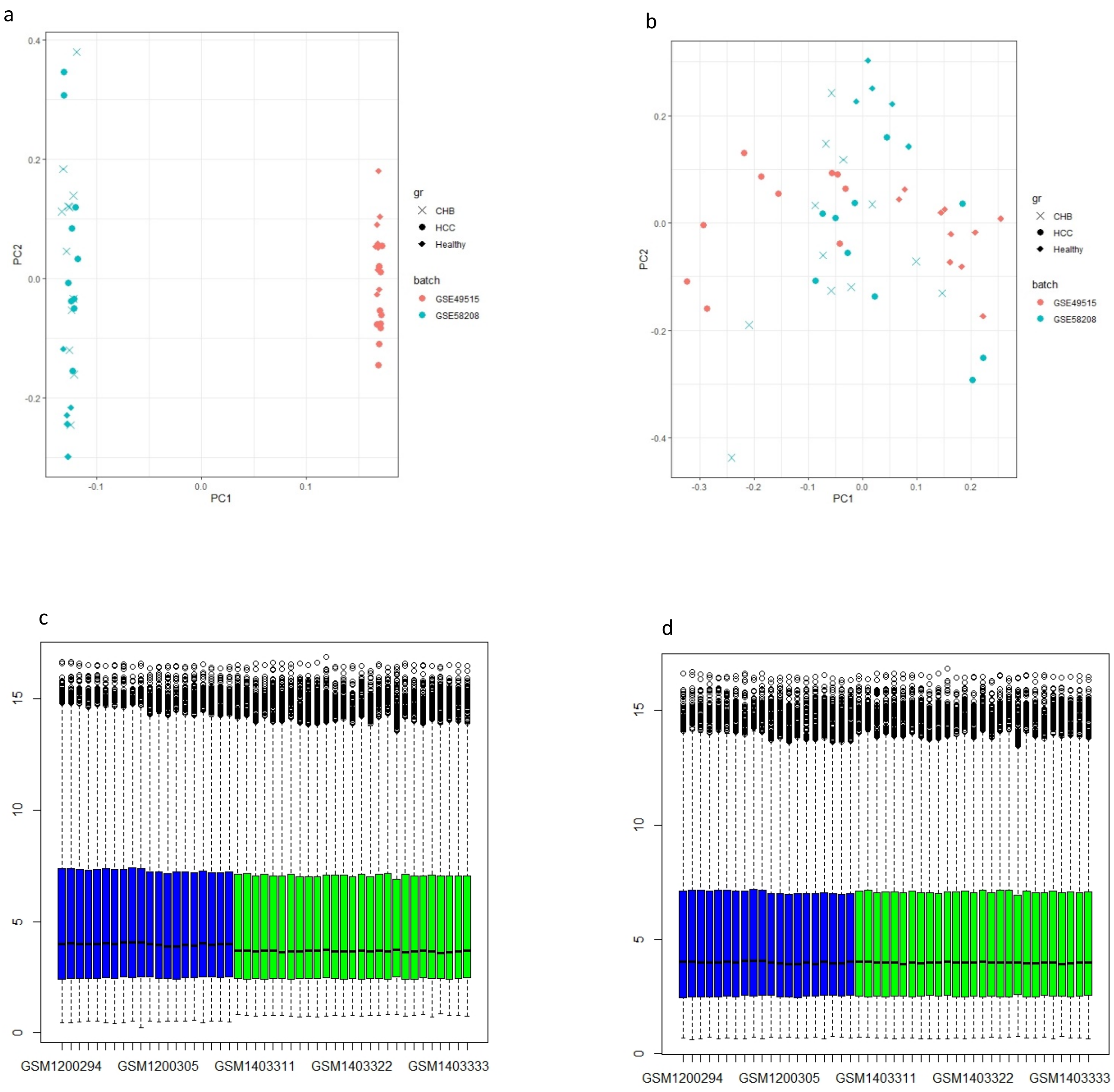

Supplementary Figure 2. Centrality parameters including betweenness, closeness, degree, eccentricity, radiality, and stress for determining hub genes a) TNF b) IL1B c) ITGA2B and d) P2RY12.

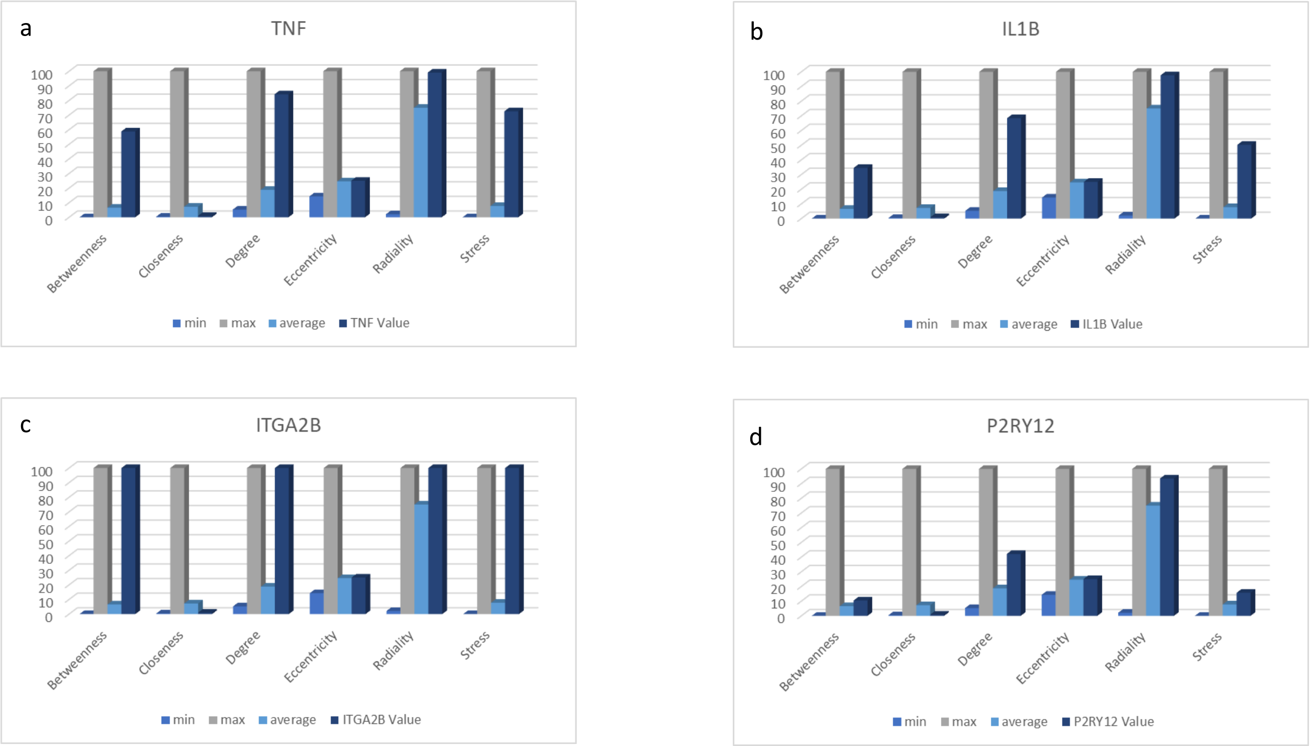

Supplementary Table 1. Accession numbers of selected datasets.

Sample Code	Data series	Group
GSM1200294	GSE49515	HCC
GSM1200295	GSE49515	HCC
GSM1200296	GSE49515	HCC
GSM1200297	GSE49515	HCC
GSM1200298	GSE49515	HCC
GSM1200299	GSE49515	HCC
GSM1200300	GSE49515	HCC
GSM1200301	GSE49515	HCC
GSM1200302	GSE49515	HCC
GSM1200303	GSE49515	HCC
GSM1403309	GSE58208	HCC
GSM1403310	GSE58208	HCC
GSM1403311	GSE58208	HCC
GSM1403312	GSE58208	HCC
GSM1403313	GSE58208	HCC
GSM1403314	GSE58208	HCC
GSM1403315	GSE58208	HCC
GSM1403316	GSE58208	HCC

GSM1403317	GSE58208	HCC
GSM1403318	GSE58208	HCC
GSM1403319	GSE58208	CHB
GSM1403320	GSE58209	CHB
GSM1403321	GSE58210	CHB
GSM1403322	GSE58211	CHB
GSM1403323	GSE58212	CHB
GSM1403324	GSE58213	CHB
GSM1403325	GSE58214	CHB
GSM1403326	GSE58215	CHB
GSM1403327	GSE58216	CHB
GSM1403328	GSE58217	CHB
GSM1403329	GSE58218	CHB
GSM1403330	GSE58219	CHB
GSM1200304	GSE49515	Healthy
GSM1200305	GSE49515	Healthy
GSM1200306	GSE49515	Healthy
GSM1200307	GSE49515	Healthy
GSM1200308	GSE49515	Healthy
GSM1200309	GSE49515	Healthy

GSM1200310	GSE49515	Healthy
GSM1200311	GSE49515	Healthy
GSM1200312	GSE49515	Healthy
GSM1200313	GSE49515	Healthy
GSM1403331	GSE58208	Healthy
GSM1403332	GSE58208	Healthy
GSM1403333	GSE58208	Healthy
GSM1403334	GSE58208	Healthy
GSM1403335	GSE58208	Healthy

Supplementary Table 2. The basic clinical characteristics of the validation cohort including HCC patients, CHB patients and healthy controls.

Variables	HCC (N=39)	CHB (N=15)	Healthy (N=24)
**Age(year)**	63.9(±4.9)	46.66(±14.27)	34.57(±10.21)
**Gender**			
Male	29(74.4%)	8 (53.3%)	16(67%)
Female	10 (25.6%)	7(46.7%)	8(33%)
**BMI**	25.66(±3.88)	25.54(±1.69)	25.18(±3.02)
**Family history**			
**Positive**	11(28.2%)	-	2(8.3%)
**Negative**	28(71.8%)		22(91.7%)
**Smoking status**			
**Ex-smoker**	12(30.8%)	5(33.3%)	0(0%)
**Smoker**	12(30.8%)	5(33.3%)	4(16.7%)
**Non-smoker**	15(38.4%)	10(66.7%)	20(83.3%)
**Drinking status**			
**Yes**	14(35.9%)	6(40%)	5(20.8%)
**No**	25(64.1%)	9(60%)	19(79.2%)
**Total Bilirubin (mg/dL)**	2.51(±1.49)	1.16 (±0.45)	
**Serum albumin (g/dL)**	3.63(±0.81)	4.48(±0.71)	
**Aspartate transaminase (AST) (IU/L)**	58.29(±39.44)	31.6(±13.03)	
**Alanine transaminase (ALT) (IU/L)**	57.29(±54.43)	32(±12.91)	
**Alkaline phosphatase (IU/L)**	320.57(±154.33)	207.25(±46.47)	
**Alpha fetoprotein test (AFP) (ng/mL)**	163.70(±39.77)	12.34(±16.37)	
**Platelet count** (10^9/L)	128.35(±82.21)	161.9(±66.73)	

Supplementary Table 3. Top features selected for each condition by multinomial LASSO feature selection method

Condition	Selected features by LASSO
**HCC**	CCNA1, ANG, RNASE2, ITGA10, TNF-α, TTC16, C8B, IL1B, C19orf33, DDX11L2, MZB1
**CHB**	FADD, TNF-α, KCNJ2, AP2B1, CISH, PLAAT1, FLJ12120
**Healthy controls**	ZNF324, DNAJB2, MAP3K7, KIAA0040, MLH3

Supplementary Table 4. Performance of machine learning on the real-life patient cohort data (HCC vs healthy) using the combination of genes presented in 4-marker panel

Panel	Sensitivity	Specificity	Accuracy	AUC
**RNASE2, MAP3K7, KCNJ2**	0.932	0.917	0.921	0957
**RNASE2, TNF-α, KCNJ2**	0.949	1	0.968	0.972

**TNF-α, MAP3K7, KCNJ2**	0.949	1	0.968	0.998
**RNASE2, TNF-α, MAP3K7**	0.974	1	0.984	1

Supplementary Table 5. Performance of machine learning models (3- to 5-marker panels) on the real-life HCC patient compared to CHB

Panel	Sensitivity	Specificity	Accuracy
**RNASE2, TNF-α, DNAJB2**	0.932	0.8	0.889
**RNASE2, TNF-α, MAP3K7, KCNJ2**	0.923	0.867	0.907
**RNASE2, TNF-α, MAP3K7, KCNJ3, DNAJB2**	0.897	0.867	0.889
**RNASE2, TNF-α, MAP3K7**	0.923	0.867	0.907

Supplementary Table 6. The oligonucleotide sequences of selected genes used in this study.

Gene name	Primer sequence 5′→ 3′	Gene name
**GAPDH**	F: CTCATTTCCTGGTATGACAACGA<br>R: CTTCCTCTTGTGCTCTTGCT	121
**RANSE2**	F: CGGAGACTGGGAAACATGG<br>R: GGAGGTTTGACATGGAGTGAG	104
**MAPK3K7**	F: TCCATTCCTTTGGTCTTTCCAG<br>R: AGCTCCTTCTTCCTTTGCTC	167
**CCNA1**	F: AGACCTGCACTTCCTGCT<br>R: GTGCCTTATTTCAGCTTCCCT	166
**TNF-α**	F: CCATGTTGTAGCAAACCCT<br>R: GGACCTGGGAGTAGATGAG	145
**KCNJ2**	F: AATTCTGGTTTGCTTTGGCTC<br>R: AGAGAACATGTCCTGTTGCTG	120
**DNAJB2**	F: GGCATCCTACTACGAGATCC<br>R: GCTTGTGCTTGTCAGACAG	182
